# Comparison of seven prognostic tools to identify low-risk pulmonary embolism in patients aged <50 years

**DOI:** 10.1038/s41598-019-55213-8

**Published:** 2019-12-27

**Authors:** Luis Jara-Palomares, Maria Alfonso, Ana Maestre, David Jimenez, Fernando Garcia-Bragado, Carme Font, Raquel Lopez Reyes, Luis Hernandez Blasco, Gemma Vidal, Remedios Otero, Manuel Monreal, The RIETE investigators, Mª Dolores Adarraga, Miguel Ángel Aibar, Jesús Aibar, Cristina Amado, Juan Ignacio Arcelus, Aitor Ballaz, Raquel Barba, Manuel Barrón, Belén Barrón-Andrés, José Bascuñana, Ángeles Blanco-Molina, Ana María Camón, Inmaculada Cañas, Cristina Carrasco, Joaquín Castro, Cristina de Ancos, Jorge Del Toro, Pablo Demelo, José Antonio Díaz-Peromingo, Raquel Díaz-Simón, Conxita Falgá, Ana Isabel Farfán, Carmen Fernández-Capitán, María del Carmen Fernández-Criado, Sandra Fernández-Núñez, Ángeles Fidalgo, Llorenç Font, Maria Angelina García, Marcial García-Morillo, Aranzázu García-Raso, Olga Gavín-Sebastián, María del Carmen Gayol, Aída Gil-Díaz, Vicente Gómez, Covadonga Gómez-Cuervo, José González-Martínez, Enric Grau, Javier Gutiérrez, Sara Gutiérrez-González, Marina Iglesias, Mª Jesús Jaras, Inés Jou, María Dolores Joya, Antonio Lalueza, Jorge Lima, Pilar Llamas, Jose Luis Lobo, Luciano López-Jiménez, Patricia López-Miguel, Juan José López-Núñez, Juan Bosco López-Sáez, Manuel Alejandro Lorente, Alicia Lorenzo, Mónica Loring, Olga Madridano, Pablo Javier Marchena, Javier Miguel Martín, Meritxell Mellado, Mª del Valle Morales, María Luisa Nieto, José Antonio Nieto, Manuel Jesús Núñez, María Carmen Olivares, José María Pedrajas, Galadriel Pellejero, Gloria Pérez-Rus, Mª Luisa Peris, José Antonio Porras, Agustina Rivas, Mª Ángeles Rodríguez-Dávila, A. Adela Rodríguez-Hernández, Carmen Mª Rubio, Pedro Ruiz-Artacho, Justo Ruiz-Ruiz, Pablo Ruiz-Sada, Joan Carles Sahuquillo, Vladimir Salazar, Ángel Sampériz, Juan Francisco Sánchez Muñoz-Torrero, Teresa Sancho, Silvia Soler, José María Suriñach, Elena Tapia, Carles Tolosa, María Isabel Torres, Javier Trujillo-Santos, Fernando Uresandi, Reina Valle, Paula Villares, Paula Gutiérrez, Fernando Javier Vázquez, Alicia Vilaseca, Thomas Vanassche, Christophe Vandenbriele, Peter Verhamme, Jana Hirmerova, Radovan Malý, Gregory Celis, Gustavo del Pozo, Estuardo Salgado, Ilham Benzidia, Laurent Bertoletti, Alessandra Bura-Riviere, Philippe Debourdeau, Dominique Farge-Bancel, Adrian Hij, Isabelle Mahé, Farès Moustafa, Sebastian Schellong, Andrei Braester, Benjamin Brenner, Inna Tzoran, Babak Sharif-Kashani, Giovanni Barillari, Franca Bilora, Cristiano Bortoluzzi, Barbara Brandolin, Eugenio Bucherini, Maurizio Ciammaichella, Francesco Dentali, Pierpaolo Di Micco, Rosa Maida, Daniela Mastroiacovo, Nicola Mumoli, Federica Pace, Roberto Parisi, Raffaelle Pesavento, Paolo Prandoni, Roberto Quintavalla, Anna Rocci, Roberta Romualdi, Carmine Sinicalchi, Antonella Tufano, Adriana Visonà, Ngoc Vo Hong, Beniamino Zalunardo, Valdis Gibietis, Dana Kigitovica, Andris Skride, Marijan Bosevski, Henri Bounameaux, Lucia Mazzolai, Joseph A. Caprini, Hanh My Bui, Khanh Quoc Pham, Abilio Reis

**Affiliations:** 1 Department of Pneumonology, Medical Surgical Unit of Respiratory Diseases, Instituto de Biomedicina de Sevilla (IBiS), Centro de Investigación Biomédica en Red de Enfermedades Respiratorias (CIBERES), Hospital Universitario Virgen del Rocío, Seville, Spain; 2 Department of Pneumonology, Complejo Hospitalario de Navarra, Pamplona, Spain; 3 Department of Internal Medicine, Hospital Universitario de Vinalopó, Alicante, Spain; 4 Respiratory Department, Hospital Universitario Ramón y Cajal, IRYCIS, Madrid, Spain; 5 Department of Internal Medicine, Hospital Universitari de Girona Dr, Josep Trueta, Gerona, Spain; 6 Department of Medical Oncology, Hospital Clínic, Barcelona, Spain; 7 Department of Pneumonology, Hospital Universitari i Politècnic La Fe, Valencia, Spain; 8 Department of Pneumonology, Hospital General Universitario de Alicante, ISABIAL, Alicante, Spain; 9 Department of Internal Medicine, Corporación Sanitaria Parc Taulí, Barcelona, Spain; 10 Department of Internal Medicine, Hospital Universitario Germans Trias i Pujol de Badalona, Barcelona, Universidad Católica de Murcia, Murcia, Spain; 11 Department of Internal Medicine, Hospital de Montilla, Córdoba, Spain; 12 Department of Internal Medicine, Hospital Clínico Universitario Lozano Blesa, Zaragoza, Spain; 13 Department of Internal Medicine, Hospital Clínic, Barcelona, Spain; 14 Department of Internal Medicine, Hospital Sierrallana, Santander, Spain; 15 Department of General Surgery, Hospital Universitario Virgen de las Nieves, Granada, Spain; 16 Department of Pneumonology, Hospital de Galdakao, Vizcaya, Spain; 17 Department of Internal Medicine, Hospital Rey Juan Carlos, Madrid, Spain; 18 Department of Pneumonology, Complejo Hospitalario San Pedro, La Rioja, Spain; 19 Centro de Investigación Biomédica de la Rioja, Fundación Rioja Salud, La Rioja, Spain; 20 Department of Internal Medicine, Hospital Universitario Infanta Leonor, Madrid, Spain; 21 Department of Internal Medicine, Hospital Universitario Reina Sofía, Córdoba, Spain; 22 Department of Internal Medicine, Hospital General de Granollers, Barcelona, Spain; 23 Department of Pneumonology, Hospital Universitario Virgen de Valme, Sevilla, Spain; 24 Department of Internal Medicine, Hospital Santa Bárbara, Puertollano, Ciudad Real Spain; 25 Department of Internal Medicine, Hospital Universitario de Fuenlabrada, Madrid, Spain; 26 Department of Internal Medicine, Hospital General Universitario Gregorio Marañón, Madrid, Spain; 27 Department of Internal Medicine, Hospital Clínico Universitario de Santiago, Santiago de Compostela, Spain; 28 Department of Internal Medicine, Hospital Universitario 12 de Octubre, Madrid, Spain; 29 Department of Internal Medicine, Hospital de Mataró, Barcelona, Spain; 30 Department of Internal Medicine, Hospital Universitario La Paz, Madrid, Spain; 31 Department of Internal Medicine, Hospital Universitario de Salamanca, Salamanca, Spain; 32 Department of Haematology, Hospital de Tortosa Verge de la Cinta, Tarragona, Spain; 33 Department of Haematology, Hospital Universitario Fundación Jiménez Díaz, Madrid, Spain; 34 Department of Haematology, Hospital Clínico Universitario Lozano Blesa, Zaragoza, Spain; 35 Department of Internal Medicine, Hospital Comarcal de Barbanza, Ribeira, A Coruña Spain; 36 Department of Internal Medicine, Hospital Universitario de Gran Canaria Dr. Negrín, Las Palmas, Spain; 37 Department of Pneumonology, Hospital Universitario Ramón y Cajal, Madrid, Spain; 38 Department of Internal Medicine, ALTAHAIA, Xarxa Assistencial de Manresa, Barcelona, Spain; 39 Department of Haematology and Hemotherapy, Hospital Lluis Alcanyis, Valencia, Spain; 40 Department of Internal Medicine, Hospital Monográfico ASEPEYO, Universidad Francisco de Vitoria, Madrid, Spain; 41 Department of Internal Medicine, Hospital Clínico Universitario de Valladolid, Valladolid, Spain; 42 Department of Internal Medicine, Hospital Cantoblanco, Madrid, Spain; 43 Department of Internal Medicine, Hospital de Figueres, Gerona, Spain; 44 Department of Pneumonology, Hospital Universitario Araba, Álava, Spain; 45 Department of Pneumonology, Hospital General Universitario de Albacete, Albacete, Spain; 46 Department of Internal Medicine, Hospital Universitario de Puerto Real, Cádiz, Spain; 47 Department of Internal Medicine, Hospital Vega Baja de Orihuela, Alicante, Spain; 48 Department of Internal Medicine, Hospital Comarcal de Axarquía, Málaga, Spain; 49 Department of Internal Medicine, Hospital Universitario Infanta Sofía, Madrid, Spain; 50 Department of Internal Medicine and Emergency, Parc Sanitari Sant Joan de Déu-Hospital General, Barcelona, Spain; 51 Department of Angiology and Vascular Surgery, Hospital del Mar, Barcelona, Spain; 52 Department of Internal Medicine, Hospital del Tajo, Madrid, Spain; 53 Department of Pneumonology, Hospital Arnau de Vilanova, Valencia, Spain; 54 Department of Internal Medicine, Hospital General Virgen de la Luz, Cuenca, Spain; 55 Department of Internal Medicine, Complejo Hospitalario de Pontevedra, Pontevedra, Spain; 56 Department of Internal Medicine, Hospital Clínico San Carlos, Madrid, Spain; 57 Department of Internal Medicine, Consorcio Hospitalario Provincial de Castellón, Castellón, CEU Cardenal Herrera University, Valencia, Spain; 58 Department of Internal Medicine, Hospital Universitario Joan XXIII de Tarragona, Tarragona, Spain; 59 Department of Internal Medicine, Hospital Alto Guadalquivir Andújar, Jaén, Spain; 60 Department of Internal Medicine, Hospital Reina Sofía, Tudela, Navarra Spain; 61 Department of Internal Medicine, Hospital Municipal de Badalona, Barcelona, Spain; 62 Department of Internal Medicine, Hospital Universitario Virgen de Arrixaca, Murcia, Spain; 63 Department of Internal Medicine, Hospital San Pedro de Alcántara, Cáceres, Spain; 64 Department of Internal Medicine, Hospital Olot i Comarcal de la Garrotxa, Gerona, Spain; 65 Department of Internal Medicine, Hospital Universitario Vall d’Hebron, Barcelona, Spain; 66 Department of Internal Medicine, Hospital General Universitario Santa Lucía, Murcia, Spain; 67 Department of Pneumonology, Hospital Universitario Cruces, Barakaldo, Vizcaya Spain; 68 Department of Internal Medicine, Hospital de Madrid Norte Sanchinarro, Madrid, Spain; 69 Department of Internal Medicine, Hospital Italiano de Buenos Aires, Buenos Aires, Argentina; 70 Department of Haematology and Haemostasis, Clínica San Camilo, Buenos Aires, Argentina; 71 Vascular Medicine and Haemostasis, University of Leuven, Leuven, Belgium; 72 Department of Internal Medicine, University Hospital Plzen, Plzen, Czech Republic; 73 Department of Cardiovascular Medicine I, Charles University in Prague, Faculty of Medicine in Hradec Kralove, University Hospital Hradec Kralove, Hradec Kralove, Czech Republic; 74 Intensive Care Unit, Hospital Clínica La Merced, Quito, Ecuador; 75 Department of Internal Medicine and Pathology, Hôpital Saint-Louis, Paris, France; 76 Department of Vascular Medicine and Therapeutics, Hôpital Nord - CHU St-Etienne, Saint-Etienne, France; 77 Department of Vascular Medicine, Hôpital de Rangueil, Toulouse, France; 78 Department of Supportive Care Oncology, Institut Sainte Catherine, Avignon, France; 79 Department of Internal Medicine, Hôpital Louis Mourier, Colombes (APHP), University Paris 7, Paris, France; 80 Department of Emergency, Clermont-Ferrand University Hospital, Clermont-Ferrand, France; 81 Department of Medical Clinic. Municipal Hospital of Dresden Friedrichstadt, Dresden, Germany; 82 Department of Haematology, Azrieli School of Medicine in Galilee, Bar-ilan University, Ramat Gan, Israel; 83 Department of Haematology and Bone Marrow Transplantation, Rambam Health Care Campus, Haifa, Israel; 84 Department of Cardiology and Heart Transplant, Masih-Daneshvari Hospital, Tehran, Iran; 85 Department of Internal Medicine, Ospedale S.Maria della Misericordia, Udine, Italy; 86 Department of Cardiovascular Sciences, Vascular Medicine Unit, University of Padua, Padua, Italy; 87 Department of Internal Medicine, Ospedale SS. Giovanni e Paolo di Venezia, Venice, Italy; 88 Department of Vascular Medicine, Ospedale Castelfranco Veneto, Castelfranco Veneto, Italy; 89 Department of Vascular Medicine, Azienda U.S.L. Di Ravenna – O.C. Di Faenza, Ravenna, Italy; 90 Department of Emergency Internal Medicine, Ospedale St. John, Rome, Italy; 91 Department of Clinical and Experimental Medicine, University of Insubria, Varese, Italy; 92 Department of Internal Medicine and Emergency Room, Ospedale Buon Consiglio Fatebenefratelli, Naples, Italy; 93 Department of Angiology, Ospedale SS. Filippo e Nicola, Avezzano, Italy; 94 Department of Medicina d´Urgenza, Ospedale San Camilo, Rome, Italy; 95 Department of Medicine 3, Azienda Ospedaliera Universitaria, Parma, Italy; 96 Regional Reference Centre for Coagulation Disorders, Department of Clinical Medicine and Surgery, Federico II, University Hospital, Naples, Italy; 97 Department of Cardiology, Ospedale Pauls Stradins Clinical University Hospital, Riga, Latvia; 98 Institute for Cardiovascular Diseases, Faculty of Medicine, Clinical Center, Skopje, Republic of Macedonia; 99 Division of Angiology and Haemostasis, Department of Internal Medicine, University Hospital of Geneva, Geneva, Switzerland; 100 Department of Angiology, Centre Hospitalier Universitaire Vaudois (CHUV), Lausanne, Switzerland; 101 Department of Medicine and Vascular Medicine, Evanston NorthShore University HealthSystem, Evanston, Illinois USA; 102 Department of Scientific research management, Hanoi Medical University Hospital, Hanoi, Vietnam; 103 Department of Cardiology, Bach Mai Hospital, Hanoi, Vietnam; 104 Head of Pulmonary Vascular Disease Unit, Centro Hospitalar do Porto, Porto, Portugal

**Keywords:** Embolism, Thromboembolism, Disease-free survival

## Abstract

In young patients with acute pulmonary embolism (PE), the predictive value of currently available prognostic tools has not been evaluated. Our objective was to compare prognostic value of 7 available tools (GPS, PESI, sPESI, Prognostic Algorithm, PREP, shock index and RIETE) in patients aged <50 years. We used the RIETE database, including PE patients from 2001 to 2017. The major outcome was 30-day all-cause mortality. Of 34,651 patients with acute PE, 5,822 (17%) were aged <50 years. Of these, 83 (1.4%) died during the first 30 days. Number of patients deemed low risk with tools was: PREP (95.9%), GPS (89.6%), PESI (87.2%), Shock index (70.9%), sPESI (59.4%), Prognostic algorithm (58%) and RIETE score (48.6%). The tools with a highest sensitivity were: Prognostic Algorithm (91.6%; 95% CI: 85.6–97.5), RIETE score (90.4%; 95%CI: 84.0–96.7) and sPESI (88%; 95% CI: 81–95). The RIETE, Prognostic Algorithm and sPESI scores obtained the highest overall sensitivity estimates for also predicting 7- and 90-day all-cause mortality, 30-day PE-related mortality, 30-day major bleeding and 30-day VTE recurrences. The proportion of low-risk patients who died within the first 30 days was lowest using the Prognostic Algorithm (0.2%), RIETE (0.3%) or sPESI (0.3%) scores. In PE patients less 50 years, 30-day mortality was low. Although sPESI, RIETE and Prognostic Algorithm scores were the most sensitive tools to identify patients at low risk to die, other tools should be evaluated in this population to obtain more efficient results.

## Introduction

The incidence and severity of acute pulmonary embolism (PE) progressively increase with patient’s age^1–3^. In the elderly, PE generally develops in patients with impaired mobility and a number of co-morbidities or the use of concomitant drugs^4–6^. In young individuals, PE frequently affects women with hormonal alterations (including pregnancy or use of contraceptives), with minor co-morbidities, or in presence thrombophilia^4–8^. Unfortunately, data on the clinical presentation, treatment, and outcomes during the course of anticoagulation in young patients with PE remain scarce. Although mortality in this population is low, the impact in terms of avoidable deaths and complications is relevant. Further, it remains unclear whether the widely available risk prediction tools (e.g., PESI, sPESI, and others) – primarily validated in older patients with a high burden of co-morbidities – do perform well in the young^9^.

In the Emergency wards, prognostic tools are commonly used to classify patients with PE into high-, medium- or low-risk categories, aimed at selecting the most appropriate management strategy at the individual level. However, a number of systematic reviews of prognostic models showed inconsistent results across the studies^10–14^. In 2016, a systematic review and meta-analysis provided evidence-based information on the validity and utility of several prognostic tools^9^, although their performances in patients aged <50 years remains unclear.

The most severe short-term complication of PE is 30-day all-cause death. The RIETE (Registro Informatizado Enfermedad TromboEmbólica) registry was established in Spain in 2001. It is an ongoing, multicenter, international observational registry of consecutive patients with objectively confirmed venous thromboembolism (VTE)^15^. The aim of this study was to compare seven currently available prognostic tools in terms of their ability to identify low-risk patients with acute PE aged <50 years.

## Methods

### Study design

We retrospectively compared seven different prognosis tools in patients aged <50 years with no-high risk acute PE. The major outcome measure was 30-day all-cause mortality. Secondary outcomes included 7-day and 90-day all-cause mortality as well as the 30-day PE-related mortality, major bleeding, and VTE recurrences rates.

### Inclusion criteria

Patients included in the RIETE registry we deemed eligible in presence of acute symptomatic PE confirmed by objective testing (pulmonary angiography, ventilation-perfusion lung scintigraphy or helical computed tomography scan). Patients were excluded if they were currently participating in a therapeutic clinical trial with a blinded study drug. The methodology of RIETE has been previously published^15^. Data were recorded from each participating hospital and submitted to a coordinating center through a secure website. Each patient was assigned with a unique identification number to maintain patient confidentiality, and data quality was regularly monitored electronically. Patients received anticoagulant treatment according to current guidelines^16–18^. All patients provided oral or written informed consent to be included in the registry, according to the requirements of ethics committees within each hospital (Authorization of clinical research ethics committee Germans Trias i Pujol and Institut Catalá de la Salud, 05122006). Researchers assessed mortality by using patient or proxy interviews and/or through review of hospital clinical records. In case of death, we performed a thorough review of medical records (accompanied by proxy interviews when necessary) to clarify the date and cause of death. Investigators were instructed to classify a death as due to PE in the following cases: 1) PE-related death confirmed on necropsy or 2) death following a clinically severe PE event (either initially or shortly after an objectively confirmed recurrent event) in the absence of any alternative diagnosis. Major bleeding was defined as bleeding occurring at high-risk anatomic locations (intracranial, intra-spinal, intraocular, retroperitoneal, intra-articular, pericardial, or intramuscular bleeding with compartment syndrome), or overt bleeding requiring a transfusion of two or more units of packed red blood cells. Confirmation of recurrent PE was documented by evidence of a novel intraluminal filling defect on CT, enlargement of a previous filling defect on CT, or evidence of a new perfusion scan defect involving >75% of a lung segment.

### Study variables and definitions

The following parameters are recorded in RIETE: patient’s baseline characteristics; clinical status (including any coexisting or underlying conditions such as chronic heart or lung disease), recent major bleedings; presence of anemia; creatinine levels; risk factors for PE; treatment received upon PE diagnosis; therapeutic outcomes; and risk factors for PE according to the ISTH criteria^19^. Recent bleeding was considered to be present in subjects who suffered a major bleeding <30 days before PE.

### Prognostic tools

Comparison of prognostic tools was performed through discrimination^20,21^. In accordance with the current guidelines^22,23^, the tools aimed at identifying low-risk PE patients were evaluated according to their sensitivity. The following prognostic tools were investigated: Geneva prognostic score (GPS)^24^, Pulmonary Embolism Severity Index (PESI)^25^, simplified PESI (sPESI)^26^, Prognostic Algorithm^27^, Facteurs PRonostiques dans l’Embolie Pulmonaire (PREP)^28^, shock index^29^, and RIETE score^30^. The prognostic tools are depicted in detail in Supplement Table 1.

### Follow-up

Patients were managed according to the clinical practice of each participating hospital and were not subjected to any predetermined intervention. For the study purpose of the study and in light of the primary outcome measure, patients were followed up for a minimum of 30 days. In addition, data at 90 days were collected for analyzing the secondary outcome. During follow-up, special attention was paid to any signs or symptoms suggestive of recurrent PE or bleeding complications. Each episode of suspected recurrent deep vein thrombosis (DVT) or PE required documented objective imaging findings. Most outcomes were classified as reported by the participating centers. However, a central adjudicating committee reviewed all outcomes reported as uncertain (less than 10% of all events).

### Statistical analysis

A descriptive analysis was performed using relative frequencies for categorical variables and means (SD) for continuous variables. We used the Student’s *t*-test and the χ^2^ test (or Fisher’s exact test where appropriate) to compare continuous or categorical variables, respectively. The 95% confidence interval (CI) for proportions was calculated using the Clopper-Pearson exact method. The discrimination of models was evaluated by the degree to which they distinguished between subjects who reached the outcome *versus* those who did not. We used the area under curve (AUC) and determined the percentage of patients deemed to be at low-risk. We assessed sensitivity, specificity, positive and negative predictive values. All calculations were performed with the SPSS statistical software, version 20 (IBM, Armonk, NY, USA). Two-sided p values < 0.05 were considered statistically significant.

## Results

**Figure 1:**
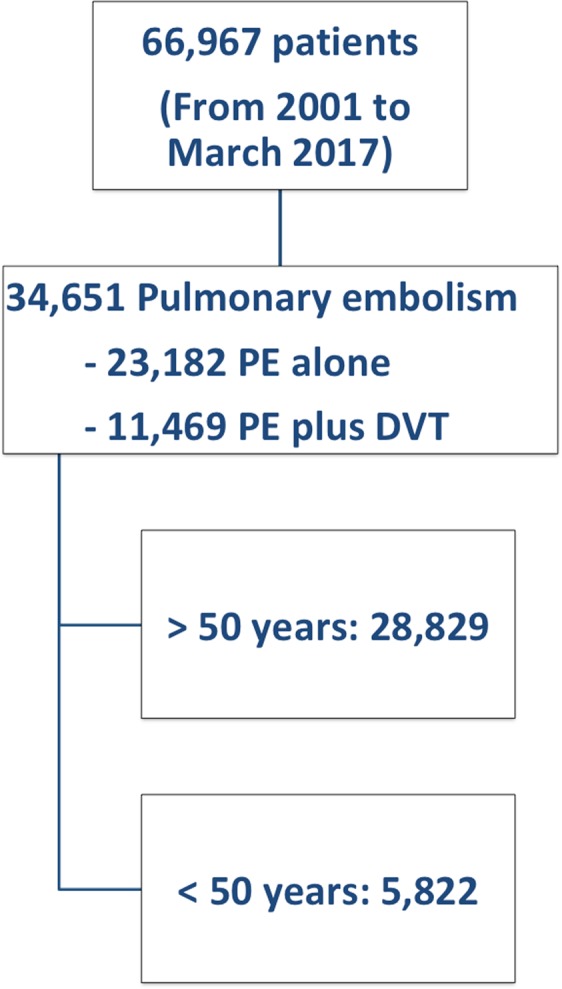
Flow diagram.

Of the 34,651 patients with acute PE enrolled in RIETE by August 2017, 5,822 (17%) were aged <50 years (Fig. 1). Patients aged <50 years were less likely to have concomitant diseases (Table 1). Moreover, they had a higher likelihood to have chest pain or tachycardia at baseline and a lower likelihood to present with syncope, hypoxemia, raised troponin levels, atrial fibrillation, right bundle branch block on the electrocardiogram, right ventricle dysfunction or raised pulmonary artery pressure levels on the echocardiogram. Finally, patients aged <50 years were more likely to score at low-risk according to PESI or sPESI. Eight of the 5,822 patients (0.14%) did not receive anticoagulant therapy. Of them, five did not start anticoagulation because they died on the same day of PE diagnosis. Three cases underwent inferior vena cava filter placement because of contraindications to anticoagulation.

**Table 1:** Clinical characteristics of the patients, according to age.

	<50 years	≥50 years
***Patients, N***	***5,822***	***28,829***
**Clinical characteristics**,		
Male sex	2,855 (49%)	13,304 (46%)
Body weight, kg/m^2^	80 ± 20	75 ± 15
Body mass index > 30 kg/m^2^ (N = 23,605)	1,217 (29%)	6,020 (31%)
**Underlying diseases**,		
Chronic lung disease	224 (3.8%)	4,716 (16%)
Chronic heart failure	70 (1.2%)	3,117 (11%)
Recent major bleeding	129 (2.2%)	684 (2.4%)
**Risk factors**,		
Active cancer	608 (10%)	7,110 (25%)
Recent surgery	916 (16%)	3,200 (11%)
Recent immobility ≥ 4 days	925 (16%)	6,476 (22%)
Pregnancy or postpartum	297 (5.1%)	11 (0.04%)
Oestrogen use	1,400 (24%)	460 (1.6%)
Recent travel	275 (4.7%)	598 (2.1%)
None of the above (unprovoked)	2,176 (37%)	13,752 (48%)
Prior VTE	696 (12%)	4,413 (15%)
**Signs or symptoms**,		
Dyspnea	4,242 (73%)	23,591 (82%)
Chest pain	3,866 (66%)	12,128 (42%)
Syncope	652 (11%)	4,418 (15%)
Abnormal mental status	90 (1.5%)	1,433 (5.0%)
Heart rate ≥ 110 bpm	1,394 (24%)	5,639 (20%)
SBP levels < 100 mm Hg	463 (8.0%)	2,326 (8.1%)
Respiratory rate > 30 pm (N = 11,555)	140 (6.8%)	860 (9.1%)
Temperature < 36 °C	116 (2.0%)	1,008 (3.5%)
Sat O_2_ levels < 90% (N = 21,796)	503 (16%)	5,961 (32%)
**Electrocardiogram**,		
Yes	4,747 (82%)	24,988 (87%)
Atrial fibrillation	35 (0.74%)	2,473 (9.9%)
Right bundle branch block	526 (11%)	4,491 (18%)
**Echocardiogram**,		
Yes	2,808 (48%)	11,929 (41%)
RV dysfunction (N = 12,714)	505 (21%)	2,503 (24%)
PAP levels > 50 mm Hg (N = 8,148)	290 (22%)	2,277 (33%)
TAPSE < 16 mm (N = 4,564)	120 (14%)	717 (19%)
**Helical CT-scan findings**,		
Subsegmental	146 (4.3%)	576 (3.4%)
Segmental	628 (18%)	2,451 (14%)
More central	1,563 (46%)	7,904 (46%)
Not reported	1,074 (31%)	6,099 (36%)
**Blood tests**,		
Anemia	1,631 (28%)	9,696 (34%)
Abnormal platelet count	369 (6.3%)	1,653 (5.7%)
CrCl levels 30-60 mL/min	116 (2.0%)	11,467 (40%)
CrCl levels < 30 mL/min	21 (0.36%)	2,208 (7.7%)
Raised troponin levels (N = 15,781)	576 (24%)	4,959 (37%)
BNP levels > 100 pg/ml (N = 2,722)	156 (44%)	1,629 (69%)

Overall, 38 patients aged <50 years (0.65%; 95% CI: 0.46–0.89%) died within the first 7 days, 83 (1.43%; 95% CI: 1.14–1.76%) within the first 30 days, and 148 (2.54%; 95% CI: 2.15–2.98%) within the first 90 days. The most common causes of death at 30 days were pulmonary embolism (39.7%) and disseminated cancer (32.4%). Moreover, 45 patients (0.77%) developed major bleeding and 63 (1.08%) had VTE recurrences within the first 30 days (55.5% recurred as PE and 44.5% as DVT).

### 30-day all-cause mortality

The proportion of patients considered to be at low risk was highest using the PREP (95.9%), GPS (89.6%) or PESI (87.2%) scores, whereas the proportion of low-risk patients who died within the first 30 days was lowest using the Prognostic Algorithm (0.2%), RIETE (0.3%), or sPESI (0.3%) scores (Table 2). The highest sensitivity was obtained using the Prognostic Algorithm (91.6%; 95% CI: 85.6–97.5%), RIETE (90.4%; 95% CI: 84–96.7%), or sPESI scores (88%; 95% CI: 81–95%; Table 3). All prognostic tools had an excellent negative predictive value. The tools that performed better in terms of specificity were PREP (96.2%; 95% CI: 95.7–96.7%), GPS (90.2%; 95% CI: 89.4–90.9%), and PESI (87.8%; 95% CI: 87–88.7%).

**Table 2:** Comparison of risk-class-specific 30-day all-cause mortality in different prognosis tools.

		Proportion (%)	Patients (n = 5,822)	Died (n = 83)	Event rate (%)
PESI^22^	Low risk	87.2	5,074	34	0.7
	High risk	12.8	748	49	6.6
sPESI^23^	Low risk	59.4	3,459	10	0.3
	High risk	40.6	2,363	73	3.1
Shock Index^26^	Low risk	70.9	4,126	32	0.8
	High risk	21.1	1,406	50	3.6
GPS^21^	Low risk	89.6	5,214	40	0.8
	High risk	10.4	608	43	7.1
Prognostic Algorithm^24^	Low risk	58	3,376	7	0.2
	High risk	42	2,446	76	3.1
PREP^25^	Low risk	95.9	5,584	66	1.2
	High risk	4.1	238	17	7.1
RIETE score^27^	Low risk	48.6	2,828	8	0.3
	High risk	51.4	2,994	75	2.5

**Table 3:** Accuracy of the prediction rule to predict 30-day mortality in different prognosis tools.

	Sensitivity	Specificity	PPV	NPV	Accuracy	AUC
	% (95% CI)	% (95% CI)	% (95% CI)	% (95% CI)	% (95% CI)	% (95% CI)
PESI^22^	59.0 (48.5–69.6)	87.8 (87–88.7)	6.6 (4.8–8.3)	99.3 (99.1–99.6)	87.4 (86.6–88.3)	0.73 (0.67–0.80)
sPESI^23^	88 (81–95)	60.1 (58.8–61.4)	3.1 (2.4–3.8)	99.7 (99.5–99.9)	60.5 (59.2–61.8)	0.74 (0.70–0.78)
Shock Index^26^	61 (50.4–71.5)	75.1 (74–76.3)	3.6 (2.6–4.5)	99.2 (99–99.5)	74.9 (73.8–76.1)	0.68 (0.62–0.74)
GPS^21^	51.8 (41.1–62.6)	90.2 (89.4–90.9)	7.1 (5–9.1)	99.2 (99–99.5)	89.6 (88.8–90.4)	0.71 (0.64–0.78)
Prognostic Algorithm ^24^	91.6 (85.6–97.5)	58.7 (57.4–60)	3.1 (2.4–3.8)	99.8 (99.6–99.9)	59.1 (57.9–60.4)	0.75 (0.71–0.79)
PREP^25^	20.5(11.8–29.2)	96.2 (95.7–96.7)	7.1 (3.9–10.4)	98.8 (98.5–99.1)	95.1 (94.5–95.6)	0.58 (0.51–0.65)
RIETE score^27^	90.4 (84–96.7)	49.1 (47.8–50.4)	2.5 (2–3.1)	99.7 (99.5–99.9)	49.7 (48.4–51)	0.70 (0.65–0.74)

### 7- and 90-day all-cause mortality and other outcomes

With regard to the prediction of 30-day PE-related mortality, the highest sensitivity estimates were obtained using the Prognostic Algorithm (90.3%; 95% CI: 79.9–100%), RIETE score (87.1%; 95% CI: 75.3–98.9%), and sPESI (83.8%; 95% CI: 70.9–96.8%; Table 4). As far as major bleeding is concerned, the highest sensitivity estimates were obtained using the RIETE (73.3%; 95%CI: 60.4–86.3%), Prognostic Algorithm (66.7%; 95% CI: 52.9–80.4%), and sPESI scores (64.4%; 95% CI: 50.5–78.4%). With regard to VTE recurrences, the highest sensitivity estimates were obtained using the RIETE (68.3%; 95% CI: 56.8–79.8%), Prognostic Algorithm (66.7%; 95% CI: 55–78.3%), and sPESI scores (65.1%; 95% CI: 53.3–76.9%). For 7- or 90-day all-cause mortality, 30-day major bleeding and 30-day VTE recurrences, the highest sensitivity estimates were obtained using the RIETE, Prognostic Algorithm, and sPESI scores (Table 5).

**Table 4:** Thirty-day PE related mortality, major bleeding and VTE recurrences of different prognosis tools.

		30-day PE-related mortality	30-day major bleeding	30-day VTE recurrences
		(n = 31)	(n = 45)	(n = 63)
		N (%)	N (%)	N (%)
PESI^22^	Low risk	14 (45.2%)	29 (64.4%)	40 (63.5%)
	High risk	17 (54.8%)	16 (35.6%)	23 (36.5%)
sPESI^23^	Low risk	5 (16.1%)	16 (35.6%)	22 (34.9%)
	High risk	26 (83.9%)	29 (64.4%)	41 (65.1%)
Shock Index^26^	Low risk	12 (38.7%)	26 (57.8%)	39 (62.9%)
	High risk	19 (61.3%)	19 (42.2%)	23 (37.1%)
GPS^21^	Low risk	18 (58.1%)	35 (77.8%)	45 (71.4%)
	High risk	13 (41.9%)	10 (22.2%)	18 (28.6%)
Prognostic Algorithm^24^	Low risk	3 (9.7%)	15 (33.3%)	21 (33.3%)
	High risk	28 (90.3%)	30 (66.7%)	42 (66.7%)
PREP^25^	Low risk	26 (83.9%)	42 (93.3%)	59 (93.7%)
	High risk	5 (16.1%)	3 (6.7%)	4 (6.4%)
RIETE score^27^	Low risk	4 (12.9%)	12 (26.7%)	20 (31.8%)
	High risk	27 (87.1%)	33 (73.3%)	43 (68.3%)

**Table 5:** Seven, 30 and 90-day all-cause mortality of different prognosis tools.

		7-day all-cause mortality	30-day all-cause mortality	90-day all-cause mortality
		(n = 38)	(n = 83)	(n = 148)
		N (%)	N (%)	N (%)
PESI^22^	Low risk	17 (44.7%)	34 (41%)	60 (40.5%)
	High risk	21 (55.3%)	49 (59%)	88 (59.5%)
sPESI^23^	Low risk	4 (10.5%)	10 (12.1%)	12 (8.1%)
	High risk	34 (89.5%)	73 (88%)	136 (91.9%)
Shock Index^26^	Low risk	11 (29%)	32 (39%)	64 (44.1%)
	High risk	27 (71.1%)	50 (61%)	81 (55.9%)
GPS^21^	Low risk	21 (55.3%)	40 (48.2%)	71 (48%)
	High risk	17 (44.7%)	43 (51.8%)	77 (52%)
Prognostic Algorithm^24^	Low risk	2 (5.3%)	7 (8.4%)	9 (6.1%)
	High risk	36 (94.7%)	76 (91.6%)	139 (93.9%)
PREP^25^	Low risk	30 (79%)	66 (79.5%)	126 (85.1%)
	High risk	8 (21.1%)	17 (20.5%)	22 (14.9%)
RIETE score^27^	Low risk	3 (7.9%)	8 (9.6%)	8 (5.4%)
	High risk	35 (92.1%)	75 (90.4%)	140 (94.6%)

## Discussion

The risk for PE progressively increases with the patient’s age. However, young patients are not uncommon, with one in every 6 cases in our cohort (17%) being aged <50 years. These patients had fewer co-morbidities, different risk factors, and different signs or symptoms at baseline compared with those aged ≥50 years. The 30-day mortality rate in our patients <50 years was low (1.43%) compared with the rate of 7.4% observed in the entire sample^9^. Despite these favorable figures, tools to identify low-risk patients should be sensitive and efficient. An improved identification of patients at risk and the use of accurate prognostic tools may pave the way to optimized management strategies (i.e., drug selection, optimal dosing, treatment settings), ultimately reducing mortality rates. The current study compared seven distinct prognostic tools in the same patient cohort. Our main results indicated that sPESI, RIETE, and the Prognostic Algorithm scores were the most efficient tools to identify PE patients aged <50 years at low risk of death within the first 30 days. Notably, these three tools also had the highest sensitivity in the prediction of 7- and 90-day all-cause mortality, 30-day PE-related mortality, 30-day major bleeding, and 30-day VTE recurrences.

Prognostic tools to identify low-risk patients with PE need to have the highest overall sensitivity and negative predictive value. Most of the tools examined in our study were effective and associated with a low event rate in patients considered at low risk. One important prerequisite is the definition of an incidence limit for a specific outcome that should be clinically relevant^9^. The correct identification of low-risk patients is paramount in real-life clinical practice. Tools characterized by a low incidence of events in the low-risk group identifies a smaller proportion of patients, being more effective but less efficient. In our study, tools with the higher proportion of patients considered to be at low-risk were PREP, Geneva, and PESI, although their sensitivity was low. The selection of the best prognostic tool in a specific population is crucial to identify subjects at low risk that may be safely discharged home or managed in an outpatient setting. Although 30-day all-cause mortality was overall low in patients aged <50 years, the social, psychological, and economic impact of such deaths is not negligible. In 2017, the HOPPE score has been specifically developed to identify low-risk patients^31^. It is characterized by a good sensitivity (96–99%) and negative predictive value (95–96%) in the prediction of 30-day mortality. The HOPPE score consists of five ordinal variables (scored as 1, 2, or 3 points, respectively), as follows: systolic blood pressure values (>120, 100 to 119, <99 mmHg), diastolic blood pressure values (>80, 65 to 79, <64 mmHg), heart rate (<80, 81 to 100, > 101 beats/min), arterial partial pressure of oxygen (>80, 60 to 79, <59 mmHg), and modified electrocardiographic score (<2, 2 to 4, >4). The following modified electrocardiographic score with adjusted variables and point values is used: tachycardia: 2 points; incomplete right bundle branch block: 1 point; complete right bundle branch block: 3 points; T-wave inversion in V1 to V3: 4 points; S1Q3T3: 4 points. The HOPPE score finally identifies three prognostic groups, as follows: low-risk: 0–6 points; intermediate-risk: 7–10 points; high-risk: 11–15 points. Although Subramanian and co-workers^31^ reported a short-term mortality of 0% (95% CI: 0–0.8%) in the low-risk group, the authors maintained that prospective validation of the HOPPE score is required before its implementation in clinical practice. Assessment of other tools or biomarkers may lead to the development of a more efficient model while maintaining a high sensitivity in the detection of low-risk patients.

The sPESI, RIETE, and Prognostic Algorithm scores are based on 6, 11, and 10 variables, respectively. The similar performances of the scores may be explained by the fact that five items are shared (Supplement Table 2) – including cancer, heart failure, pulse ≥110 beats per minute, systolic blood pressure <100 mmHg, and arterial blood oxygen saturation <90%. The Prognostic Algorithm and sPESI also have age in common, whereas the Prognostic Algorithm and RIETE score share common chronic renal disease. For the sake of simplicity, the use of sPESI may be recommended. One of the most interesting findings was that PESI, a consistently validated tool, did not perform well in this patient population. Although patients deemed at low risk according to PESI had a low 30-day mortality rate (0.7%; 95% CI: 0.5–0.9%), the sensitivity of the tool was low as well (59%; 95% CI: 48.5–69.6%). We speculate that this may stem from the influence of patient’s age on the score calculation. Accordingly, age is treated as a quantitative variable in PESI.

This work has several limitations. First, the design of RIETE does not randomize patients to different strategies or drugs, although quality-control audits are periodically implemented. We believe that our registry may provide relevant real-life data in a large number of patients observed outside the rigorous and controlled conditions of clinical trials. As such, it may be helpful to identify risk factors for clinical outcomes in an unselected patient population. Second, it is likely that patients who died early after PE (i.e., within 2–3 days) were not included in the cohort (because of lack of informed consent or death in emergency room). Third, the attribution of deaths to PE may be difficult owing to the lack of a validated definition based on broadly accepted criteria. Although causes of death were investigated by a thorough review of medical records, an overestimation of PE-related mortality cannot be ruled out^32^.

This study has two strengths. First, we simultaneously analyzed seven different prognostic tools in a large population of over 5,000 patients. This approach overcomes the caveats of heterogeneity in meta-analyses or propensity scores matching. Second, this study evaluated not only 30-day all-cause mortality but also other short-term complications of PE (i.e., PE-related death, VTE recurrences, and bleedings).

## Conclusion

We compared seven prognostic tools to identify which was the most sensitive to identify patients aged <50 years with acute PE at low risk for 30-day mortality. The most performing tools were sPESI, RIETE, and Prognostic Algorithm. Because the mortality rates in our population were low, more efficient tools or biomarkers are required to improve the prognostic categorization of this patient group. Compared with elderly cases, PE patients aged <50 years have a different profile, with less co-morbidities, different risk factors, and different signs and symptoms.

## Supplementary information

Prognostic tools
