# Supplementary material for: Comparison of seven prognostic tools to identify low-risk pulmonary embolism in patients aged <50 years
Source: Sci Rep. 2019 Dec 27;9:20064. doi: 10.1038/s41598-019-55213-8 (PMC6934558; doi:10.1038/s41598-019-55213-8)
Supplement: Supplementary file 1 — Prognostic tools [file 41598_2019_55213_MOESM1_ESM.pdf]

Original Article

Title: Comparison of seven prognostic tools to identify low-risk pulmonary embolism in patients aged <50 years

Running head: Prognostic tools in patients <50 years with PE

Authors: Luis Jara-Palomares[1], MD, Maria Alfonso[2], MD, Ana Maestre[3], MD, PhD, David Jimenez[4], MD, PhD, Fernando Garcia-Bragado[5], MD, PhD, Carme Font[6], MD. PhD, Raquel Lopez Reyes[7], MD, Luis Hernandez Blasco[8], MD. PhD, Gemma Vidal[9], Remedios Otero[1], MD, MD, Manuel Monreal[10], MD, PhD and the RIETE investigators*

Affiliations

[1] Department of Pneumonology, Medical Surgical Unit of Respiratory Diseases, Instituto de Biomedicina de Sevilla (IBiS), Centro de Investigación Biomédica en Red de Enfermedades Respiratorias (CIBERES), Hospital Universitario Virgen del Rocío, Seville, Spain
[2] Department of Pneumonology, Complejo Hospitalario de Navarra, Pamplona, Spain
[3] Department of Internal Medicine, Hospital Universitario de Vinalopó, Alicante, Spain
[4] Respiratory Department, Hospital Universitario Ramón y Cajal, IRYCIS, Madrid, Spain
[5] Department of Internal Medicine, Hospital Universitari de Girona Dr, Josep Trueta, Gerona, Spain
[6] Department of Medical Oncology, Hospital Clínic, Barcelona, Spain
[7] Department of Pneumonology, Hospital Universitari i Politècnic La Fe, Valencia, Spain
[8] Department of Pneumonology, Hospital General Universitario de Alicante, ISABIAL, Alicante, Spain
[9] Department of Internal Medicine, Corporación Sanitaria Parc Taulí, Barcelona, Spain
[10] Department of Internal Medicine, Hospital Universitario Germans Trias i Pujol de Badalona, Barcelona, Universidad Católica de Murcia, Spain

*A full list of the RIETE investigators is provided in the appendix

PROGNOSTIC TOOLS

PESI (Pulmonary embolism severity index) (1)

	PESI
Age	Age, in year
Male sex	10
Cancer	30
Heart failure	10
Chronic lung disease	10
Pulse ≥ 110 min	20
Systolic blood pressure < 100 mmHg	30
Altered mental status	60
Arterial blood oxygen saturation < 90%	20
Respiratory rate ‡ 30 $^{min-1}$	20
Temperature < 36 C	20
I: Very low ≤ 65<br>II: Low 66–85<br>III: Intermediate 86–105<br>IV: High 106–125<br>V: Very high ‡ 126<br><br>I–II: Low ≤ 85<br>III–IV: High > 85	

Simplified PESI (sPESI) (2)

	sPESI
Age > 80 years	1
Cancer	1
Chronic cardiopulmonary disease	1
Pulse ≥ 110 min	1
Systolic blood pressure < 100 mmHg	1
Arterial blood oxygen saturation < 90%	1
Low risk: ≤ 0<br>High risk: ≥1	

SHOCK INDEX (3)

Shock index (SI): heart rate/systolic blood pressure (HR/SBP)

Low-risk: < 0.9
High-risk ≥ 0.9

GENEVA PROGNOSTIC SCORE (GPS) (4)

	GPS
Cancer	2
Heart failure	1
Previous deep vein thrombosis	1
Systolic blood pressure < 100 mmHg	2
PaO2 < 60 mmHg	1
Deep vein thrombosis shown by ultrasound	1
Low risk: ≤ 2<br>Low risk: ≥3	

PROGNOSTIC ALGORITHM (5)

	Prognostic Algorithm
Age > 70 years	1
Cancer	1
Heart failure	1
Chronic lung disease	1
Chronic renal disease	1
Cerebrovascular disease	1
Pulse ≥ 110 min	1
Systolic blood pressure < 100 mmHg	1
Altered mental status	1
Arterial blood oxygen saturation < 90%	1
Low risk: 0<br>High risk ≥ 1	

RIETE SCORE (6)

	RIETE score
Chronic heart failure	1
Recent immobility ≥ 4 days	1
Cancer without metastases	1
Cancer with metastases	2
Recent major bleeding	2
CrCl 30-60 ml/min	1
CrCl, 30 ml/min	3
Platelet count <100,000/ml or >450,000/ml	1
Systolic blood pressure < 100 mmHg	1
Heart rate ≥ 110 bpm	1
SpO2 levels <90%	1
Low risk: 0<br>High risk ≥ 1	
Definition of abbreviations: bpm = beats/min; CI = confidence interval; CrCl = creatinine clearance levels; PE = pulmonary embolism; SBP = systolic blood pressure.	

PREP (7)

	PREP
Altered mental status*	10
Shock cardiogenic on admission	6
Cancer	6
BNP (ng/L)	
• < 100	0
• 100-249	1
• 250-499	2
• 500-999	4
• ≥ 1,000	8
RV/LV ratio	
• 0.2-0.49	0
• 0.5-0.74	3
• 0.75-1.00	5
• 1.00-1.25	8
• ≥ 1.25	11
Low-risk: ≤ 6	
Intermediate-high-risk: ≥6	
*: Altered mental state was defined as disorientation, stupor, or coma	

References

1. Aujesky D, Obrosky DS, Stone RA, et al. A prediction rule to identify low-risk patients with pulmonary embolism. *Arch Intern Med*. 2006;166(2):169-175.
2. Jiménez D, Aujesky D, Moores L, et al.; RIETE Investigators. Simplification of the pulmonary embolism severity index for prognostication in patients with acute symptomatic pulmonary embolism. *Arch Intern Med*. 2010;170(15):1383-1389.
3. Toosi MS, Merlino JD, Leeper KV. Prognostic value of the shock index along with transthoracic echocardiography in risk stratification of patients with acute pulmonary embolism. *Am J Cardiol*. 2008;101(5):700-705.
4. Wicki J, Perrier A, Perneger TV, Bounameaux H, Junod AF. Predicting adverse outcome in patients with acute pulmonary embolism: a risk score. *Thromb Haemost*. 2000;84(4):548-552.
5. Aujesky D, Obrosky DS, Stone RA, et al. Derivation and validation of a prognostic model for pulmonary embolism. *Am J Respir Crit Care Med*. 2005;172(8):1041-1046.
6. Maestre A, Trujillo-Santos J, Riera-Mestre A, et al.; RIETE Investigators. Identification of Low-Risk Patients with Acute Symptomatic Pulmonary Embolism for Outpatient Therapy. *Ann Am Thorac Soc.* 2015;12(8):1122-1129.
7. Sanchez O, Trinquart L, Caille V, et al. Prognostic factors for pulmonary embolism: the prep study, a prospective multicenter cohort study. *Am J Respir Crit Care Med.* 2010;181(2):168-173.
